# Development of nanochitosan‐based active packaging films containing free and nanoliposome caraway (*Carum carvi*. L) seed extract

**DOI:** 10.1002/fsn3.2025

**Published:** 2020-11-18

**Authors:** Parisa Homayounpour, Nabi Shariatifar, Mahmood Alizadeh‐Sani

**Affiliations:** ^1^ Department of Food Science and Technology Islamic Azad University Damghan Branch Damghan Iran; ^2^ Food Safety and Hygiene Division Department of Environmental Health School of Public Health Tehran University of Medical Sciences Tehran Iran; ^3^ Halal Research Center of Islamic Republic of Iran Tehran Iran

**Keywords:** active packaging, caraway seed extract, nanochitosan film, nanoliposome, nanostructured biopolymer

## Abstract

The biocompatible active films were prepared based on nanochitosan (NCh) containing free and nanoliposome caraway seed extract (NLCE). The produced films were characterized by physico‐mechanical, barrier, structural, color, antimicrobial, and antioxidant properties. The average particle size of NLCE was 78–122 nm, and the encapsulation efficiency (EE%) was obtained 49.87%–73.07%. Nanoliposomes with the lowest size and the highest encapsulation efficiency were merged with the film samples. NCh/CE3% and NCh/NLCE3% films had higher stability compared to other films and showed the highest antimicrobial activity (3.68 mm inhibition) and radical quenching capacity (51%), respectively. Likewise, biodegradable active films containing nanoliposomes had lower antimicrobial potential and higher antioxidant capacity than films containing free extract with similar concentration. The Fourier‐transform infrared spectroscopy (FTIR) results revealed new interactions between NCh and nanoliposomes. Scanning electron microscopy (SEM) investigation also exhibited a homogenous structure and nearly smooth surface morphology with a good dispersion for NCh/NLCE films. Despite an increase in yellowness (*b value*) and a decrease in whiteness (*L value*) index, the incorporation of nanoliposomes within the NCh films improved the mechanical flexibility (from 10.2% to 15.05%) and reduced water vapor permeability (WVP) (from 14.2 × 10^–12^ g/m·s·Pa to 11.9 × 10^–12^ g/m·s·Pa). Today, due to the growing trend toward natural ingredients, the use of nanoparticles derived from plant derivatives has expanded in the food industry owing to their antimicrobial and antioxidant properties.

## Graphical abstract

The article describes the development of eco‐friendly nanostructured chitosan films that might be used as biodegradable packaging materials to replace plastics. In particular, it shows that the physical, mechanical, barrier, antioxidant, and antimicrobial properties of nanocomposite films can be modulated by adding essential oil nanoliposomes into chitosan matric.

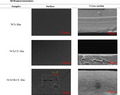

## 1 INTRODUCTION

Edible packaging films based on natural materials have been widely used to preserve food products. Accordingly, environmentally friendly films are known as “green consumerism or biodegradable packaging.” These bio‐based packaging, especially in the composite state, show acceptable barrier and protection properties against external factors such as water vapor, gases, aroma, and microorganisms (Alizadeh‐Sani et al., 2020; Bajer et al., 2020; Omrani Fard et al., 2020). Using this method is important to assure safety, promote food quality, and extend the shelf life of food products (Yu et al., 2018). Typically, biodegradable composite films are constructed from proteins (e.g., casein, whey, zein, soy collagen, wheat gluten, gelatin, keratin, and egg albumen), carbohydrates (e.g., alginate, pectin, cellulose, starch, agar, and chitosan), and lipids (e.g., acyl‐glycerol, fatty acids, and waxes) together or with other nanomaterials to improve structural and physical characteristics, mechanical resistance, and barrier (water vapor, light, gases and aroma) properties (Alizadeh‐Sani et al., 2019; Bagheri et al., 2019; Dehghani et al., 2018). Therefore, these approaches have led to the introduction of active nanocomposite packaging films in the food industry, which increase the shelf life of food by controlled release of antimicrobial and antioxidant compounds (Alizadeh‐Sani et al., 2020).

Chitosan (a linear polysaccharide obtained from deacteylation of chitin consisting of 1,4‐linked glucosamine and N‐acetylglucosamine) is a natural biopolymer, which is used to design active and edible packaging (Rodrigues et al., 2020; Tran et al., 2020). This polymer has a cationic structure and is the second most important polysaccharide in nature after cellulose. Chitosan has a variety of properties such as nontoxic, antimicrobial, antifungal, and antioxidant activities. It also has the property of forming film and coating, which has been used in recent years to increase the shelf life and improve the quality of various foods (Kerch, 2015) including vegetables (Devlieghere et al., 2004; Kerch, 2015), eggs (Kim et al., 2008), cheese (Duan et al., 2007), meats (Ouattar et al., 2000; Pabast et al., 2018), and fish (Kerch, 2015).

Hydrophobic compounds such as lipids, essential oils, and extracts are usually added to the polymeric compounds to improve film properties (Imran et al., 2012). *Carum carvi* L., which is known as Persian cumin (*Carum carvi*), is a biennial plant in the family Apiaceae. The caraway plant is native to northern and central Europe, and Central Asia (Iran, Pakistan, India, Turkmenistan) (Darougheh et al., 2014; Seo et al., 2009). The caraway seeds are used as a spice in food due to their flavor. Since ancient times, essential oil, extract, and seed of caraway have been used traditionally in yogurt, dough, cheese, meat, rice, candy, and nonalcoholic and alcoholic beverages (Darougheh et al., 2014). In medicine, caraway seed is used in medicine as antispasmodic, antiflatulence, antidysmenorrhea, antidiarrheal, and appetizer (Keshavarz et al., 2013; Seo et al., 2009). Extract and essential oil of caraway seed contain, antimicrobial, antioxidant, fungicidal, insecticidal, and diuretic properties (Foroumand et al., 2019; Raal et al., 2012). In recent years, nanoencapsulation of essential oils and plant extracts have been used to protect the physical and chemical stabilization of active ingredients and to control the release of active ingredients (Chinnachamy et al., 2014). Due to these advantages, caraway seed extract (CE) is used as a suitable material in the production of edible film.

Notwithstanding the good antimicrobial and antioxidant nature of essential oils (EO)/or extracts, their direct usage is often limited due to their intense aroma and making change in the organoleptic properties of food. Consequently, incorporating them into the structure of films has been known as an attractive approach to overcome these problems. Hence, nanotechnology and nanoencapsulation of essential oils/or extracts by using suitable polymeric micro‐ and nanoparticle systems could be one of the potent approaches which have been suggested to overcome these deficiencies (Gibis et al., 2012; Pabast et al., 2018). Recently, liposomes have been extensively studied for their potential ability to encapsulate lipophilic substances, for example, essential oils/or extracts. These phospholipid vesicles have a significant ability to entrap both hydrophilic and hydrophobic molecules. As a result, nanoliposomes are considered as an ideal carrier for bioactive components (Almasi et al., 2016; Pabast et al., 2018).

According to the various beneficial properties of caraway seed extract, this study was designed to produce edible NCh‐based films incorporated with the free and nanoliposomes caraway seed extract. In the following, the biodegradable films were characterized by using the physico‐mechanical, barrier, structural, color, antimicrobial, and antioxidant properties.

## 2 EXPERIMENTAL SECTION

### 2.1 Material

Chitosan (50,000–190,000 WM) with a deacetylation degree of 95%, glycerol with >97% purity, 2,2‐Diphenyl‐1‐picrylhydrazyl (DPPH), and chloroform were purchased from Sigma Aldrich. Phospholipid (L‐alpha‐lecithin granular with 99% purity) was purchased from Across, USA. Cholesterol (95% purity), dichloromethane, and methanol were obtained from Merck. Deionized water was used for all stages of the study. The other materials for film testes, nanoliposomes tests, and chemical sample tests were purchased from Merck.

### 2.2 Preparation of caraway seed extract

Fifty grams of caraway seeds was powdered and added to 150 ml of distilled water and stirred with bath sonication at 40°C for 20 hr and then was filtered using Whatman No.3, and then centrifuged at 5,000 *g* for 15 min. The extract was dried in oven at 40°C for 24 hr and finally, aliquot and stored in a sterile glass container at 4°C until use (Yadav & Agarwala, 2011).

### 2.3 Preparation of nanochitosan

NCh was prepared according to the method developed by de Moura et al. At first, chitosan was dissolved in malic acid solution (0.5 v/v%) and stirred by magnetic stirrer for 2 hr (Velp, Scientifica, Europe) at 500 rpm. Subsequently, 0.2 mmol of potassium peroxide sulfate was added under continuous stirring at 70°C for 3 hr, followed solution was cooled with in an ice bath. The suspension was centrifuged at 10977 *g* for 30 min, and the supernatant was discarded. The residual was dissolved with HPLC grade water for further use [22].

### 2.4 Preparation of nanoliposomes

Nanoliposomes with caraway seed extract were prepared using cholesterol and soy lecithin by thin films hydration according to Kirby and Gregoriadis, (1984) method (Kirby & Gregoriadis, 1984). Initially, four different ratios of lecithin and cholesterol (60:0, 50:10, 40:20, and 30:30 v/v) were prepared and placed in dichloromethane/methanol (1:1 v/v) in a 100 ml flask. 60 mg of caraway seed extract was dissolved in 10 ml of methanol and mixed well. Subsequently, the organic solvent was evaporated using a rotary evaporator at 30°C and then a thin film was created on the balloon walls. The thin film hydrated with 15 ml distilled water and homogenized at 44800 *g* for 10 min by Ultra‐Turrax homogenizer (Heidolf) at a temperature above the gel liquid transition temperature (Tc) of the amphiphiles. Finally, liposomal suspensions were sonicated (Sonics & Materials vibracell) for 6 min (6 cycles of 1 min each with 1 min rest) to produce monolayer nanoliposomes (Pabast et al., 2018).

### 2.5 Preparation of nanochitosan‐based films

NCh‐based films (3% w/v) were formulated by casting method. Nanoliposomes and caraway seed extract were added in two concentrations (1.5% and 3% v/v), separately, into the film solution and mixed at 300 rpm for 1 hr. Then, 40 ml of the film solution was poured in a Petri dish (10 cm‐diameter) and was dried for 48 hr at 25°C and then kept in a desiccator (RH = 50%) for other experiments.

### 2.6 Nanoliposomes characteristics

#### 2.6.1 Particle size determination

The particle size distribution and the mean diameter of nanoliposomes were measured using dynamic light scattering (DLS) technique by means of a Particle Size Analyzer (SALD 2101).Samples were prepared with 1:100 dilution using distilled water, and measurements were performed at 25°C in three replicates (Almasi et al., 2016; Pabast et al., 2018). Results are presented as a mean diameter of the liposome suspension (z‐average) by the polydispersity index (PDI) which evaluates the width of size distribution.

#### 2.6.2 Zeta potential

The electrophoretic mobility of the nanoliposomes, also referred to zeta potential, can be used to determine the charge of the surface at the particle interface in an aqueous dispersion. The zeta potential of the solution was evaluated using a Malvern zeta sizer Nano ZS (Malvern Instruments, Worcestershire) at 25°C. Prior to the measurement, the sample was diluted using distilled water to a concentration of 0.01% (Wu et al., 2016).

#### 2.6.3 Encapsulation efficiency

The encapsulation efficiency (EE%), which indicates the percentage of the total target compound(s) in liposomes, is an important parameter associated with liposome characterization. The phenolic compounds of the CE trapped in the liposomes were investigated according to a previously reported method by Gibis et al. (2012). The liposomes were isolated using an ammonium filter, and then, 3 ml of the 15% w/w triton X‐100 was intentionally added to the sample for detecting the polyphenols according to the Folin–Ciocalteu method. 1 ml of 2% sodium carbonate and 200 ml of the Folin–Ciocalteu reaction were added to 10 ml of the sample, which was then centrifuged for 5 min at 161 *g*. After 30 min reaction time, spectrophotometric measurements were performed at 750 nm using a UV–visible spectrophotometer (Pharmacia Biotech Ultra‐spec 2000) (Rajabi et al., 2019). EE (%) can be given as follows:

(1) $$\text{Encapsulation}\;\text{efficiency}\left(\%\right)=\frac{\text{Encapsulated}\;\text{phenolic}\;\text{content}}{\text{Total}\;\text{phenolic}\;\text{content}}*100$$

### 2.7 Physico‐mechanical properties of films

#### 2.7.1 Thickness

Films thickness using IP 65 micrometer (Mitutoyo Manufacturing) with an accuracy of 0.0001 mm was measured. The thickness of the films was taken at five random points on the films. Mean thickness values for each sample were calculated and used in water vapor permeability and mechanical properties calculation.

#### 2.7.2 Water vapor permeability

Using a modified version of standard ASTM E96‐00 method, the water vapor permeability (WVP) of the films was evaluated at 25°C (ASTM, 2000) (Acevedo‐Fani et al., 2015). Methyl methacrylate test cups (inner diameter of 3 cm, outer diameter of 4.5 cm and depth of 2.0 cm) were used. The cups were filled with 6 ml distilled water, and then, circular specimens of the films were placed on the cups using a rubber O‐ring cap. Then, 3 cm in diameter of the film was exposed. Glass cups were exposed to glass containers with hermetic coatings containing saturated magnesium chloride solution (MgCl_2_.6H_2_O), and conditions of 33% relative humidity were provided at 25°C. The weight of the cups was recorded at 60 min intervals within 6 hr. The magnitude of the cup weight changes over time and was used to obtain the slope (m_1_) of the curve at the steam transfer rate g/s. WVP was calculated using the following equations (Acevedo‐Fani et al., 2015):

(2) $$\text{WVTR}=m_{1}/A$$

(3) $$\text{WVP}\left(g\cdot m/m^{2}\cdot s\cdot Pa\right)=\left(L\times \text{WVTR}\right)/\left(P_{i}‐P_{a}\right)$$

Here, *A* = The exposed film area (m^2^), Pi = The vapor pressures of saturated air at 25°C, *Pa* = The vapor pressures of saturated air with RH 33% at 25°C, *L* = The average film thickness (m).

#### 2.7.3 Mechanical properties

The tensile strength (TS) and elongation to break (ETB) of the films were measured according to the standard test method of ASTM D882–10 (ASTM, 2010) using a tensile analyzer (Zwick/Roell Model FR010) at 25°C ± 2°C. After conditioning for two days at an RH = 50%, three film samples were cut and inserted between the clamps. The separation of the primary clamp and the cross‐head speed was 50 mm and 5 mm/min, respectively (Hadi Almasi et al., 2010; Cheng et al., 2015).

#### 2.7.4 Color determination

The color of film surface was determined using a colorimetric Hunter‐lab (CR 300, Konica Minolta). The results were averaged from six readings across each sample, and the total color difference (ΔE) was calculated according to the following equation:

(4) $$\Delta E=\sqrt{{\left(\Delta L*\right)}^{2}+{\left(\Delta a*\right)}^{2}+{\left(\Delta b*\right)}^{2}}$$

Δ*E* is total color difference, and ΔL*, Δa*, and Δb* are the differences between a sample color parameter and the color parameter of a standard (L* = 91.5, a* = 1.01, b* = −2.20) used as the film background as a white.

#### 2.7.5 Fourier‐transform infrared spectroscopy (FTIR)

The intermolecular and intramolecular interactions of film components were determined using FTIR spectroscopy. In this study, FTIR spectroscopy was performed with KBr pellet using a spectrometer (Shimadzu 4100, Thermo Nicolet) with a resolution of 3.2 cm and a scan range of 400 cm^−1^ to 4,000 cm^−1^ for changing functional groups. For sample preparation, three drops of film solution were put onto a KBr pellet and dried slowly and then were placed into a KBr pellet of about 1 mm thickness (Gibis et al., 2012).

#### 2.7.6 Analysis of microstructural

Distribution of the nanoliposomes in the biopolymer network was assessed by the surface and cross‐sectional area of the scanning electron microscopy (SEM) (DSM 940 A, Zeiss) images of the film samples. The frozen sample (5 mm × 5 mm) was fixed on the support using aluminum paste and carbon at an angle of 90° to the surface, which allowed observation of the cross section of the film and subsequently vacuum‐coated with gold‐plated sputum (DST1, Nanostructured Coating Company). Indeed, the film was fixed with carbon and metalized with evaporated gold in a Blazers SCD 050 sputter coater (Balzers Union AG, Liechtenstein) to grant electrically conductive properties. The surface and cross‐sectional area of the films was visualized by a SEM with a 10 KV excitation voltage of 10 mm (Pabast et al., 2018).

#### 2.7.7 Antimicrobial activity

The films antimicrobial activity was assessed by using agar diffusion method against *Staphylococcus aureus* (ATCC13565). An inhibition zone assay was conducted with add 100 μl of inoculate containing ~10^5^ cfu/ml of tested strain and streaked out over the surface of Muller–Hinton agar plates. Various films were cut into 6 mm diameter disks and then transferred on the inoculated agar (López‐Mata et al., 2013). Incubation was done at 37°C for 18–24 hr, and then, the inhibition zone was measured by a digital caliper.

#### 2.7.8 Antioxidant activity

The ability of the hydrogen atom to lose phenolic compounds is assessed by the degree of discoloration of the purple solution of 2,2‐diphenyl‐1‐picrylhydrazyl (DPPH) in methanol. In fact, DPPH is used as a stable radical which combine with the extract and then the antioxidant activity of was calculated before and after encapsulation. 1 ml of the film solution in different dilutions was mixed with 2 μl of standard methanol solution of DPPH (1.5%). The mixture was then kept at room temperature for 30 min at dark. The results of the adsorption solution were measured at 517 nm by using a UV–visible spectrophotometer (Pharmacia Biotech Ultra‐spec 2000, UK). Following equation is used to measure DPPH radical scavenging capacity (Bettaieb Rebey, Jabri‐Karoui, et al., 2012).

(5) $$\text{DPPH}\;\text{scavenging}\;\text{capacity}\left(\%\right)=\left(\left(A_{0}‐A_{1}\right)/A_{0}\right)\times 100$$

Here, *A*_0_: Control absorbance, *A*_1_: Sample absorbance.

### 2.8 Statistical analysis

The results are presented as mean ± *SD*, and the *p* value of less than .05 was considered as significant. One‐way analysis of variance (ANOVA) was used to investigate the mean separation was applied by Duncan`s multiple ranges test using SPSS software (IBM SPSS Statistics, version 21).

## 3 RESULTS AND DISCUSSION

### 3.1 Size and zeta potential

**Figure 1:**
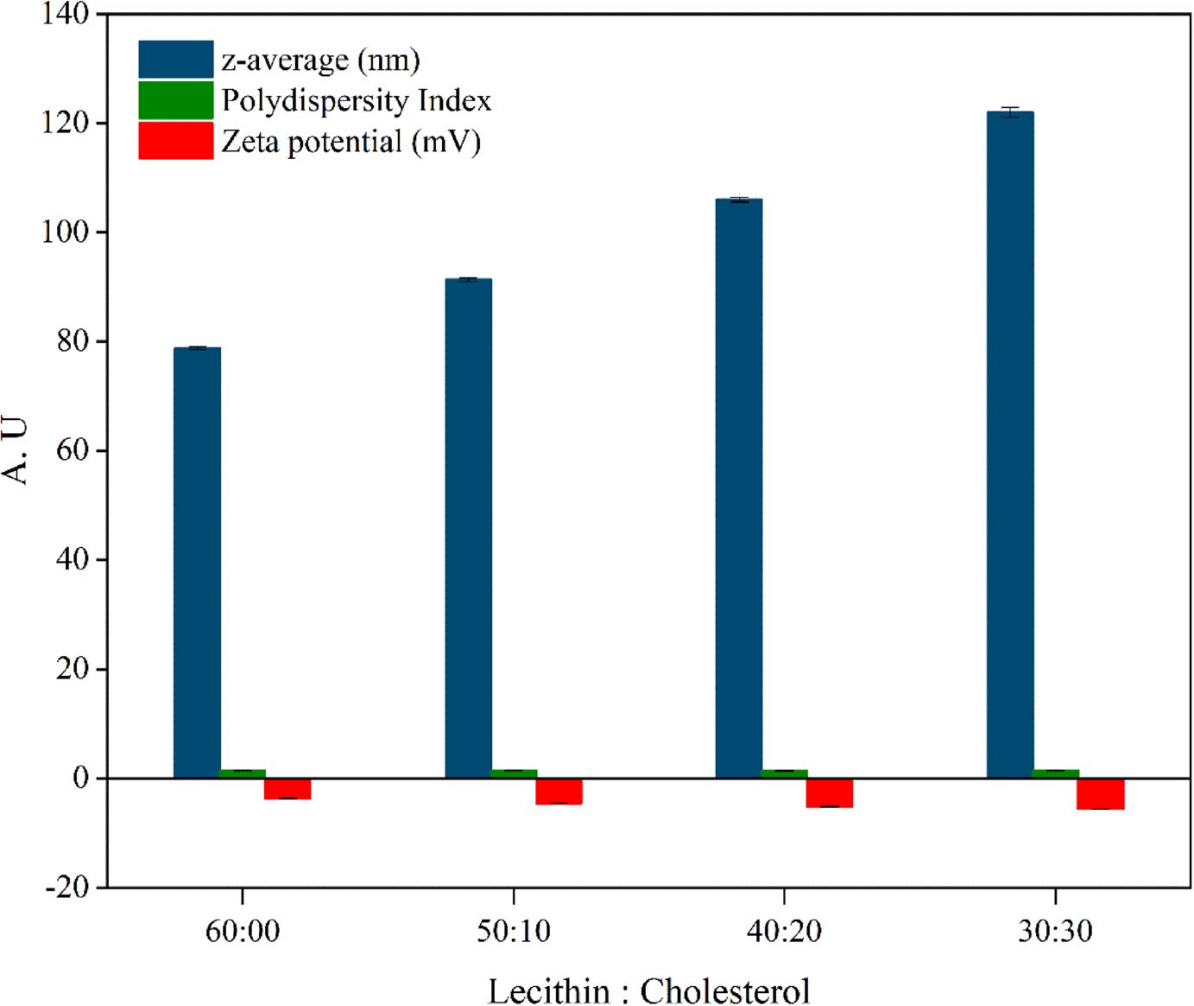
Characterization of nanoliposomes (NL)

The stability of the nanoliposomes and the potential release of the entrapped bioactive compounds into the liposome are affected by their size. Figure 1 shows the particle sizes, PDI, and zeta potentials of four differently formulated nanoliposomes. Different nanoliposomes with different formulations exhibited an average size and size distribution of 78.8–122 nm and 0.80–0.91, respectively. The z‐potential values were in the range of −3.72 and −5.63 mV. The zeta potential was increased with increasing the amount of cholesterol content in nanoliposomes formula.

The results showed that the different levels of cholesterol in four formulations did not significantly influence the particle size and polydispersity of the nanoliposomes (*p* > .05). Our results were in agreement with those of a previously conducted study Mohammadi et al. (2014) in case of vitamin‐loaded nanoliposomes. In contrast, a previous study Alexander et al. (2012) investigated the discrepancy among the reported cholesterol effects on the z‐average diameter that would be related to the variation of methods in case of liposome preparation. Figure 1 presents the zeta potential variation of nanoliposomes with different phosphatidylcholine (lecithin) and cholesterol ratios. The terminal groups on the phospholipids are in charge of the negative values of the zeta potential in this study, which is in agreement with that observed in a similar study (Monteiro et al., 2014). Furthermore, the absolute value of the negative charge of liposomes significantly increased with an increase in the proportion of cholesterol. In previously conducted studies, the changes in zeta potential can be attributed to the strong interaction between the methyl groups (originating from the choline group of lecithin) and the hydroxyl group of cholesterol via an anomalous‐type hydrogen bond (da Silva Malheiros et al., 2012; Wu et al., 2015). Furthermore, these changes are proportional to the cholesterol content in the lecithin vesicles.

### 3.2 Encapsulation efficiency

**Figure 2:**
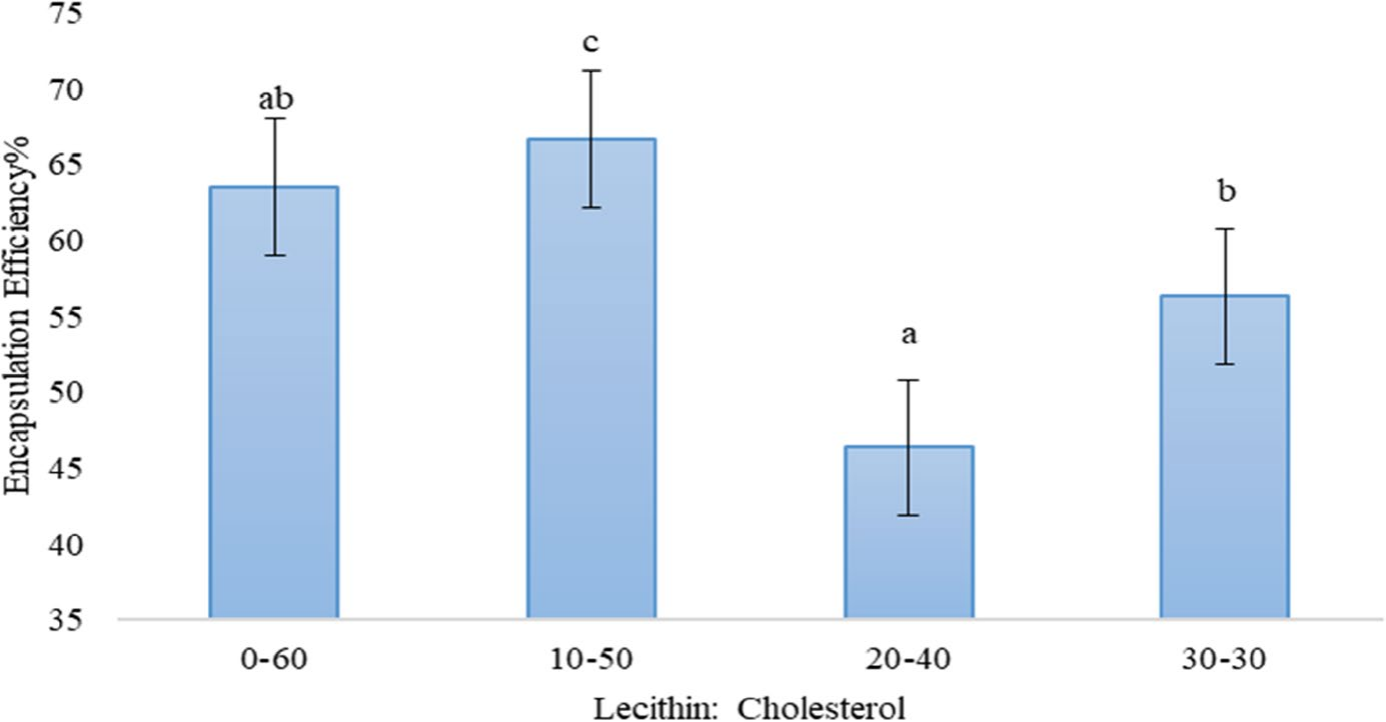
Encapsulation Efficiency (EE%) for the nanoliposomes produced via different treatments

From an economical point of view, encapsulation efficiency (EE) is one of the key factors assessed in nanoliposomes. In this study, caraway content was applied as indicator. As shown in Figure 2, the EE ranged from 46.35% to 66.63% for 20/40 and 10/50 (Lecithin: Cholesterol), respectively. Some studies reported significantly higher encapsulation efficacy than this study. Soliman et al. (2013) and Aguilar et al. (2015) reported about 90% for encapsulation of essential oils (thyme and clove oil) and sunflower, respectively. The results also showed that the ratio of 10/50 Lecithin: Cholesterol had the highest EE% and decreased with increasing lectin content. So that the ratio of 20/40 Lecithin: Cholesterol had the lowest EE%. These phenomena usually attributed to the oil nature of caraway and cholesterol which acted as nanoliposomes structure (Rajabi et al., 2019).

### 3.3 Thickness, tensile strength, and elongation to break

Films thickness varied from ~103 to ~118 μm (Table 1). The presence of extract and nanoliposomes in the films resulted in higher thicknesses due to the increase in dry matter. NCh/NLCE3% film was the thickest film especially when compared with the plain NCh film (*p* < .05). The thickness of NLCE films was higher than CE films, and also, a dose‐dependent trend was observed.

**Table 1:** Tensile strength (TS), elongation to break (ETB), water vapor permeability (WVP), and thickness of nanochitosan‐based films

Sample	TS (MPa)	ETB (%)	WVP (×10^–12^ g/m·s·Pa)	Thickness (mm)
NCh	36.39 ± 0.43^e^	10.21 ± 0.45^a^	14.2 ± 0.66^b^	0.103 ± 0.003^a^
NCh/CE1.5%	23.29 ± 0.87^c^	14.79 ± 0.54^c^	13.5 ± 0.51^c^	0.105 ± 0.001^a^
NCh/CE3%	20.54 ± 1.11^a^	17.97 ± 1.01^d^	12.4 ± 0.25^a^	0.107 ± 0.006^b^
NCh/NLCE1.5%	32.21 ± 0.76^d^	13.33 ± 0.48^b^	12.7 ± 0.43^a^	0.111 ± 0.009^c^
NCh/NLCE3%	22.45 ± 0.70^b^	15.05 ± 0.33^c^	11.9 ± 0.36^d^	0.118 ± 0.010^d^

Note

The data are presented as mean ± standard deviation. Any two means in the same column followed by the same letter are not significantly (*p* > .05) different from Duncan's multiple range tests.

Abbreviations: CE, Caraway seed extract; NCh, Nanochitosan; NLCE, Nanoliposome caraway seed extract; Data are the mean of triplicate.

The TS and ETB of films are presented in Table 1. The NCh film had the highest (36.39 MPa) and the lowest ETB (10.21%). NCh/CE3% film had the lowest TS )20.54 MPa) and the highest ETB (17.97%). The combination the free CE with film led to in lower TS and higher ETB compared to control film. The tensile strength of NCh films was also significantly reduced by combining NLCE (*p* < .05). NLCE films with 1.5% and 3% CE had a significant effect on ETB. In general, films containing nanoliposomes have better tensile strength than treatments containing free CE. The CE molecules of free can play a plasticizer role in the biopolymer matrix and thus reduce the interactions of macromolecules as a result, it can lead to a decrease in TS (strength) and an increase in ETB (flexibility) (Bonilla et al., 2012). The effect of nanoliposomes on mechanical properties can also be explained by the discontinuity created in the polymer matrix by the lecithin, which caused a change in polymer chains when lipid components are present, and a result, it will lead to a weak mechanical response (Haghju et al., 2016).

### 3.4 Water vapor permeability

To prevent food depletion, films used as packaging or coating should control the transfer of moisture from the product to the environment, so WVP edible films can used for this purpose (Ma et al., 2008). The WVP for NCh film sample was 14.2 × 10^–12^ g/m·s·Pa (Table 1). Generally, the effects of free or liposomal CE on WVP of NCh film were significant (*p* < .05). Compared to other films, NCh/NLCE3% film showed the lowest WVP (11.9 × 10^–12^ g/m·s·Pa). However, it was significantly different that other CE‐loaded film samples and pure NCh film. WVP is carried out by the hydrophobic part of the films, and the permeability depends on its lipophilic hydrophobicity. Therefore, the presence of lipid compounds in the film structure due to the increase of swelling causes the water barrier property which creates water vapor resistance through the film (Acevedo‐Fani et al., 2015; Hernandez, 1994). Therefore, the presence of nanoliposomes probably creates a difficult path for the passage of water molecules. Similar to our findings, a significant reduction in WVP has been reported with a combination of inactive nanoparticle biopolymer films (Imran et al., 2012; Ma et al., 2016; Wu et al., 2015).

### 3.5 Scanning electron microscopy

**Figure 3:**
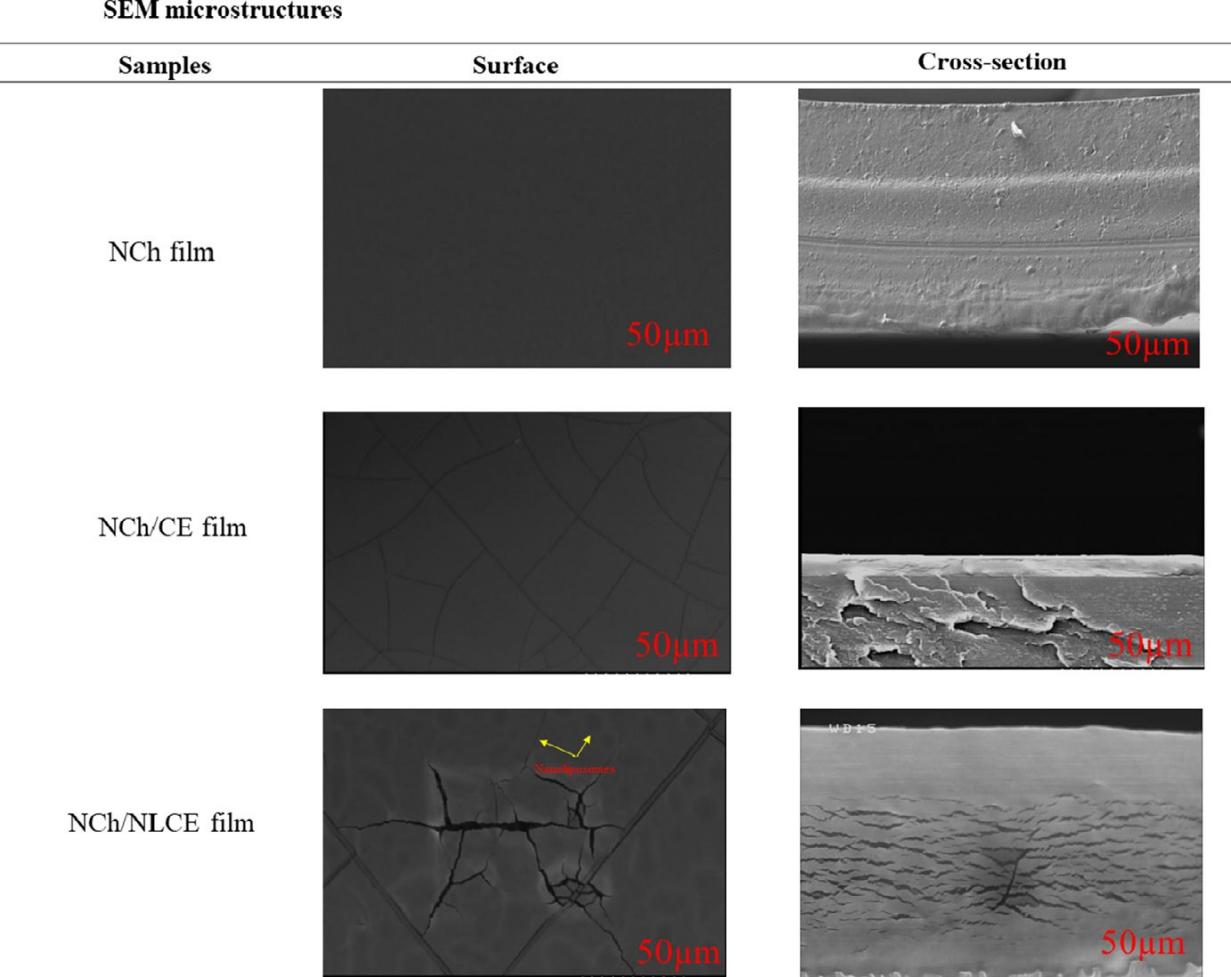
SEM micrograph of NCh, NCh/CE, and NCh/NLCE films (CE, Caraway seed extract; NCh, Nanochitosan; NLCE, Nanoliposome caraway seed extract)

The microstructure of the nanoliposome‐based films with extract was investigated to gain some insights into the organization of nanoparticles along the biopolymer matrix and its possible effect on the film properties. The SEM images in Figure 3 correspond to the surface of NCh films containing CE and NLCE. As a general trend, the structure of the biodegradable films incorporated with caraway seed extract and nanoliposomes was more difficult than NCh films. Large‐scale increases in the presence of extract have been previously observed by other authors (Norajit et al., 2010; Shojaee‐Aliabadi et al., 2013). The NCh control films were compact and had a smooth surface, without cavities or cracks. Similar results have been obtained by other researchers (Pabast et al., 2018). Although the hydrophobic nature of the extract (NCh/CE or NCh/NLCE) could affect the hydrophilic–hydrophobic properties of the films, physical factors had a major influence on the rate of water vapor passage through the films. As shown in the Figure 3, the films containing caraway seed extract had a spongy structure. The surface and cross section of the films were covered with numerous cavities. It appears that the sponge‐like structure created in these films was due to the disruption of the regular structure of the polymer chains caused by the compounds in the extract. This phenomenon has significantly increased the rate of water vapor passage through edible films. In concordance with the images observed in Figure 3, films formed in this study evidenced a structural arrangement of NCh molecules and CE governed by their chemical affinity. The addition of essential oils resulted in a relatively heterogeneous structure in the films, which the oil droplets are trapped in a continuous, uniform polysaccharide network. The size of the oil droplets in the films increased with increasing essential oil concentration. In water–oil emulsions with higher lipid levels, this is because higher lipids result in increased droplet collisions and eventually increase clotting and coagulation rates (Shojaee‐Aliabadi et al., 2013). In the study of chitosan films by Shen and Kamdem (2015), a more homogeneous structure was observed in accordance with our study results. The essential oil droplets were trapped in the integrated carbohydrate network, and the number and size of lipid droplets increased with increasing essential oil concentration (Shen & Kamdem, 2015).

### 3.6 FTIR spectroscopy

**Figure 4:**
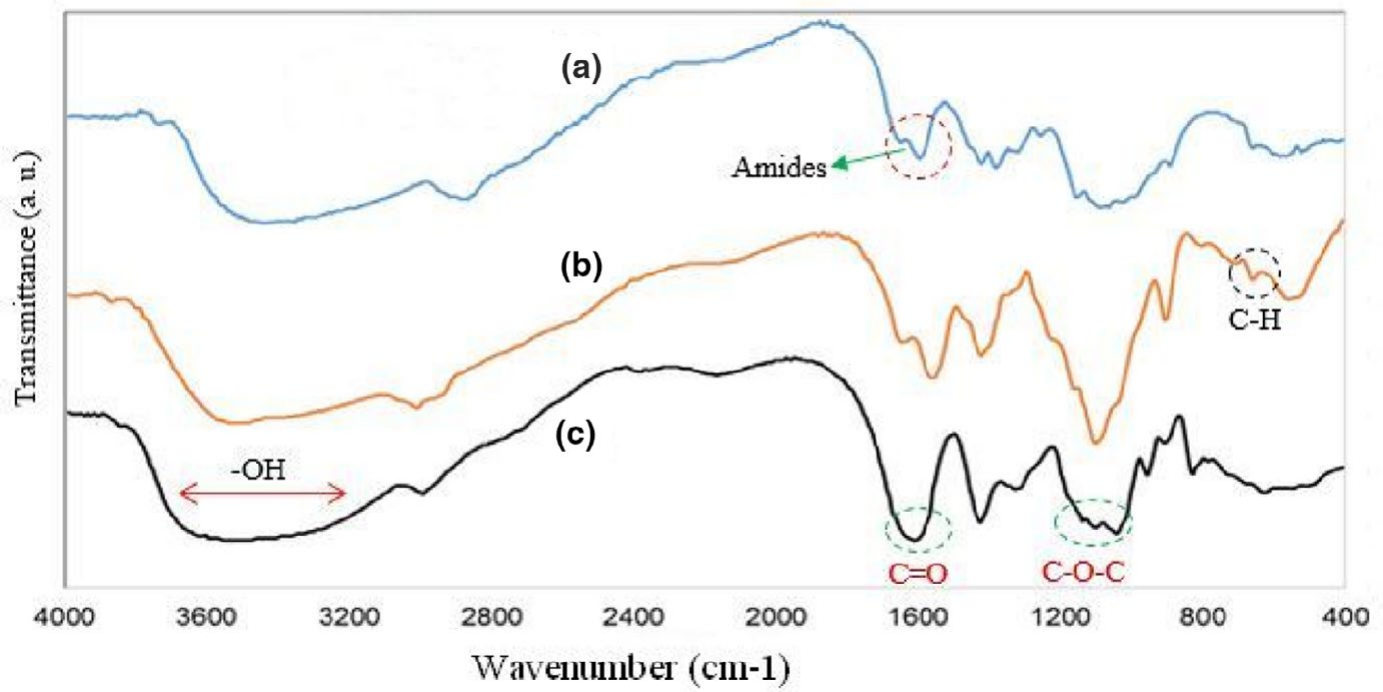
FTIR spectra of (a): NCh, (b): NCh/CE, (c): NCh/NLCE films (CE, Caraway seed extract; NCh, Nanochitosan; NLCE, Nanoliposome caraway seed extract)

Infrared spectroscopy (FTIR) of NCh film solutions incorporated with free and NLCE shown in Figure 4. Film containing free extract showed less intensity at 3,455.45 cm^−1^ that the characteristic absorption of O‐H and N‐H group stretching vibrations, and 2,931.26 cm^−1^ group aliphatic C‐H stretching vibration, CH_2_ stretching vibration at 3,078.57 cm^−1^, 3,020.76 cm^−1^ (CH_3_ group stretching vibrations), 1731.15 cm^−1^ group C = O stretching of amide I, 1664.02 cm^−1^ NH of amide II stretching vibration, 1,108.31 cm^−1^ C_3_‐OH group stretching vibrations, and 1,029.58 cm^−1^ C_6_‐OH group bending vibrations, when compared to film with nanoliposomes. In addition, a new peak for films containing free extract observed at about 1,337 cm^−1^, which can be related to phenolic compounds of the caraway seed extract. Indeed, peaks around 1,000–1,800 cm^−1^ might be ascribed to the stretching of C = O, ‐C = C‐C = O, ‐C = C‐ [(in‐ring) aromatic] and ‐C‐C‐ [(in‐ring) aromatic] found in the phenolic components. The similar peak also observed for films incorporating with extract nanoliposomes. The reason for the higher intensity of 1,337 cm^−1^ peak for NCh/NLCE films, compared to the free extract samples, is probably due to the synergistic effect of the extract and the lecithin phenolic compounds that have been extracted along with the phospholipids in the extract loaded nanoliposomes, so it can be conducted that there was some interactions between NH_2_ or OH groups of chitosan and nanoliposomes. Haghju et al. (2016) reported that the findings on FT‐IR analysis show that some new interaction has appeared between chitosan matrix and tested nanoliposomes. Also, considerable interactions between film matrix (gelatin/chitosan) and betanin nanoliposomes were observed in Amjadi et al. (2020) study. Our results were in agreement with Haghju et al. and Amjadi et al. studies (Amjadi et al., 2020; Haghju et al., 2016).

### 3.7 Color properties

**Figure 5:**
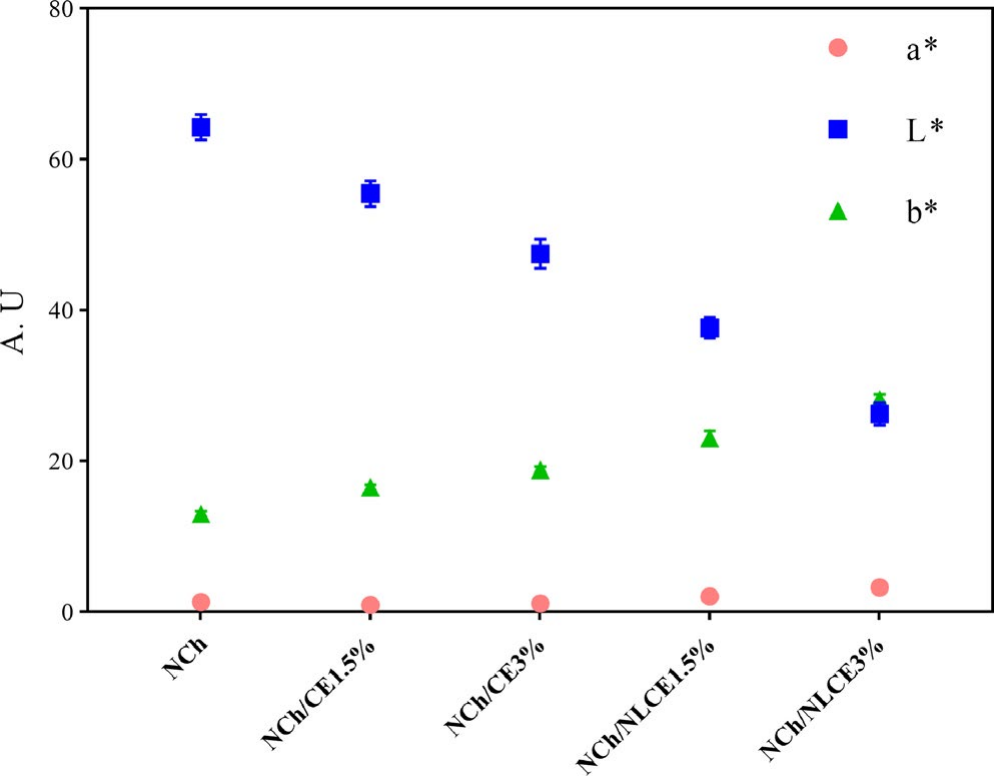
L*, a*, and b* color parameter, in different group of films (CE, Caraway seed extract; NCh, Nanochitosan; NLCE, Nanoliposome caraway seed extract)

The color characteristics of films are important factors in selecting a product for the customer, depending on the type of compound used and the process used in the film forming. Figure 5 shows the average colorimetric parameters for the films. These results were determined on the basic parameters of L*, a*, and b* that indicate (L* = Lightness value 0 = black to 100 = Whiteness), b* = yellowing (+) or blue (−) and a* = red (+) or green (−). As can be seen in Figure 5, there is a significant difference between the mean data in terms of parameter L* between the NLCE and CE film and with different percentages of extract. NCh has the highest L* value, but after adding of the nanoliposomes and extract, the films whiteness (L* value) gradually decreased. The reason for these differences is due to the color of the extract, which affects the level of brightness and uniformity of the film's surface structure by creating opacity. The data for parameter b* for NCh/CE and NCh/NLCE films were significantly higher than NCh film. Because with the addition of extract and liposome, the yellowness (b*) index increased. These phenomena may be due to forming an uneven surface on the film surface during drying, which is the CE of the film that normally accumulates on the film surface and causes surface heterogeneity. However, at low concentrations of extract, these changes are not significant.

### 3.8 Antimicrobial activity of nanohitosan‐based films

The antimicrobial activity of NCh‐based films was investigated against *Staphylococcus aureus* as a gram‐positive bacteria (Table 2). This is the first report on the antimicrobial activity of NCh films blended with caraway seed extracts against *S. aureus*. NCh‐based films were effective against *S. aureus* with a different percentage of free extract and nanoliposomes. Inhibition zone diameters of NCh, NCh/CE1.5%, NCh/CE3%, NCh/NLCE1.5%, and NCh/NLCE3% films were ranged in 1.73, 2.34, 3.68, 2.15, and 2.87 mm, respectively. *S. aureus* is often resistant to conventional antibiotic drugs due to its ability to withstand stiffness and the expression of active flow pumps; however, in the present study, chitosan film blended with caraway seed extract has shown good activity against *S. aureus*. The mechanism of action of caraway seed extract may be related to changes in the internal pH of the cell that result in bacterial cell membrane damage (Gonelimali et al., 2018; Zivanovic et al., 2005). Also, NCh/CE3% film indicated the greatest antimicrobial activity (3.68 mm) than the other composite films, and NCh control film showed lower inhibition zone than other films (1.73 mm). In the study of antimicrobial and physicochemical properties of chitosan film mixed with different essential oils on Bologna sausage and in vitro, Zivanovic et al. (2005) exhibited antimicrobial effects against *E. coli* and *L. monocytogenes*. Chitosan film reduced the number of *Listeria* to 2 logarithms and decreased the amount of growth by adding essential oils, especially *Origanum majorana* (Zivanovic et al., 2005). In another study, Amjadi et al. (2020) designed a functional nanocomposite film containing betanin nanoliposomes based on gelatin/chitosan for fresh beef preservation. They described that the nanocomposite films incorporated with betanin nanoliposomes revealed high antibacterial activity against *S. aureus* and *E. coli* (Amjadi et al., 2020).

**Table 2:** Antioxidant capacity and antimicrobial activity of the Nanochitosan‐based active films against *Staphylococcus aurous*

Sample	Antioxidant activity (%)	Inhibition zone diameter (mm)
NCh	20.14 ± 1.25^a^	1.73 ± 0.03^a^
NCh/CE1.5%	39.60 ± 0.90^b^	2.34 ± 0.02^b^
NCh/CE3%	48.36 ± 0.90^c^	3.68 ± 0.04^c^
NCh/NLCE1.5%	43.00 ± 0.50^d^	2.15 ± 0.02^b^
NCh/NLCE3%	51.00 ± 1.40^e^	2.87 ± 0.02^d^

Note

The data are presented as mean ± standard deviation. Any two means in the same column followed by the same letter are not significantly (*p* > .05) different from Duncan's multiple range tests.

Abbreviations: CE, Caraway seed extract; NCh, Nanochitosan; NLCE, Nanoliposome caraway seed extract; Data are the mean of triplicate.

### 3.9 Antioxidant activity

Antioxidant activity of NCh‐based films with different concentrations of CE and NLCE was performed by DPPH assay. Results revealed that antioxidant activity was increased in active films containing CE and NLCE compared to pure NCh films and the difference was significant (*p* ≤ .05) (Table 2). The highest antioxidant activity was observed between formulated films with CE3% (48.36%) and NLCE3% (51%). The results displayed that NCh film had the lowest antioxidant activity (20.14%) due to its low solubility. Indeed, NH_2_ amine groups react with free radicals and led to the production of stable macromolecules and ammonium NH_3_ and inhibit from absorbing H_2_ by NH_2_ (Cheng et al., 2015). So that it can be said the primary amino groups of NCh cause antioxidation mechanism of chitosan (Jridi et al., 2014). Meanwhile, the ability to give hydrogen atom and entrapping DPPH radical inside the films was defined as antioxidation mechanism. On the other hand, antioxidant activity in films loaded with extract and liposomes is attributed to the presence of polyphenolic compounds in the extract (Haghju et al., 2016; López‐Mata et al., 2013; Shojaee‐Aliabadi et al., 2013). The antioxidant effect of chitosan was also reported by another research, and degree of deacetylation of chitosan can be effective on this parameter (Kamkar et al., 2013; Pastor et al., 2013). Likewise, Bettaieb Rebey, Bourgou, et al. (2012) and Ghasemi Pirbalouti et al. (2013) also in their study reported antioxidant activity in cumin treated edible film (Bettaieb Rebey, Bourgou, et al., 2012; Ghasemi Pirbalouti et al., 2013). Amjadi et al. (2020) designed a functional betanin nanoliposomes‐incorporated gelatin/chitosan nanocomposite film for fresh beef preservation. They reported that the nanocomposite films incorporated with nanoliposomes exhibited high antioxidant properties versus DPPH radical (Amjadi et al., 2020).

## 4 CONCLUSION

The extensive cross‐linking in the films containing caraway seed extract and NLCE resulted in more antimicrobial activity, high antioxidant activity, improve the mechanical resistance, and reduced WVP. SEM images showed that the microstructure of the films containing the extract differed in terms of the cavity, surface shape, and compression pattern in freely and nanoliposomes form. Also, FTIR spectroscopy results revealed new interactions between NCh and nanoliposomes. Particularly, in some properties such as changes in thickness, percentage of elongation to break, some microstructural properties, and antimicrobial and antioxidant activity, films containing caraway seed extract (especially with CE3%) showed better results. Today, due to the growing trend toward natural ingredients, the use of nanoparticles derived from plant derivatives has expanded in the food industry owing to its antioxidant and antimicrobial properties.

## CONFLICT OF INTEREST

The authors state that there was no conflict of interest.
